# Relief of Cadmium-Induced Intestinal Motility Disorder in Mice by *Lactobacillus plantarum* CCFM8610

**DOI:** 10.3389/fimmu.2020.619574

**Published:** 2020-12-10

**Authors:** Yang Liu, Jiangping Wu, Yue Xiao, Qing Liu, Leilei Yu, Fengwei Tian, Jianxin Zhao, Hao Zhang, Wei Chen, Qixiao Zhai

**Affiliations:** ^1^ State Key Laboratory of Food Science and Technology, Jiangnan University, Wuxi, China; ^2^ School of Food Science and Technology, Jiangnan University, Wuxi, China; ^3^ National Engineering Research Center for Functional Food, Jiangnan University, Wuxi, China; ^4^ Wuxi Translational Medicine Research Center and Jiangsu Translational Medicine Research Institute Wuxi Branch, Wuxi, China; ^5^ Beijing Innovation Centre of Food Nutrition and Human Health, Beijing Technology and Business University (BTBU), Beijing, China

**Keywords:** *Lactobacillus plantarum*, cadmium, intestinal motility, proteomic analysis, neurotransmitter, cellular stress

## Abstract

Cadmium (Cd) is a toxic metal inducing a range of adverse effects on organs including liver and kidneys. However, the underlying molecular mechanisms of Cd-induced intestinal toxicity through dietary intake is poorly studied. This study evaluated the toxic effects of Cd on intestinal physiology and confirmed the effectiveness of the protective mechanism of the probiotic *Lactobacillus plantarum* CCFM8610 against chronic Cd toxicity. After treatment with Cd, the HT-29 cell line was subjected to iTRAQ analysis, which revealed that changes in the proteomic profiles after Cd exposure were related to pathways involved in the stress response and carbohydrate metabolism. The results of an animal trial also indicated that 10 weeks of Cd exposure decreased the fecal water content and contractile response of colonic muscle strips in mice, and delayed the excretion time of the first black feces. *L. plantarum* CCFM8610 treatment provided protective effects against these Cd-induced intestinal motility dysfunctions by recovering the levels of neurotransmitters, including substance P, acetyl cholinesterase, vasoactive intestinal peptide, 5-hydroxytryptamine, calcitonin gene-related peptide, and nitric oxide, and suppressing the cellular stress response in mice (e.g., the inhibition of mitogen-activated protein kinase pathways). The administration of this probiotic was also observed to reduce Cd levels in the tissues and blood of the mice. Our results suggest a newly identified protective mechanism of probiotics against Cd toxicity that involves the recovery of intestinal motility and increase in fecal cadmium excretion.

## Introduction

Cadmium (Cd) is a heavy metal widely distributed in the environment. Once Cd has accumulated in the host, it can induce a range of adverse effects on the liver, kidney, brain, bone, and reproductive systems (1–4). The mechanisms of Cd toxicity have been well studied, including its competitive interference with the metabolism of essential divalent metals such as zinc and manganese, cytotoxicity induced by the abnormal production of reactive oxide species, and disruption of protein structures by the binding of Cd to sulfhydryl groups (5–7).

For the human population, food and drinking water are the main sources of Cd exposure (8). Research has shown that after oral intake, a major fraction of Cd (approximately 60%) is absorbed by the gut as a result of mucociliary clearance and subsequent ingestion (9). Although a number of studies have demonstrated that the liver, kidneys, and lungs are the main targets of long-term Cd exposure (10), reports on the toxic effects of this heavy metal on the gut are relatively limited. Animal studies have shown that the oral administration of Cd induce pathological changes in gastric mucosa and intestinal villi (11). Cross-sectional epidemiological studies have reported Cd exposure to be accompanied by increased incidence of intestinal abnormalities such as constipation, vomiting, nausea, anorexia, and indigestion (12, 13). Cd exposure has also been shown to significantly affect intestinal motility, which delays fecal Cd excretion and exacerbates intestinal Cd absorption (11). Several studies have indicated that Cd may strongly stimulate the intestinal mucosa, inhibit digestive enzymes, disrupt gut barrier function, and adversely impact gut immunity (11, 14, 15). However, the underlying molecular mechanisms of Cd-induced intestinal toxicity are not thoroughly understood.

A number of animal trials showed that probiotics, including *Lactobacillus rhamnosus* GG, *L. rhamnosus* LC705 (16), and a cocktail of Lactobacillus and Bifidobacterium strains (17) can alleviate Cd toxicity *in vivo*. The protective effects of these strains may be due to their Cd-binding ability, which promotes fecal excretion of this metal (18). In our previous studies, *L. plantarum* CCFM8610, a probiotic isolated from fermented Chinese food, showed good Cd-binding and Cd-tolerance abilities *in vitro* (19). Subsequent animal and human trials confirmed that the administration of this probiotic can significantly reduce the Cd burden *in vivo* (20, 21). Interestingly, we found this strain to also markedly alleviate Cd-induced constipation in mice (22). Cd exposure may be detrimental to host health through increasing intestinal permeability and inducing dysfunctions in gut microbiota (23). A recent study showed that Cd treatment could markedly decreased the microbial richness and the abundance of short chain fatty acid-producing bacteria in the gut of mice (24). Furthermore, it has been reported that the intestinal bacterial species diversity of mice decreased gradually with the increase of Cd concentration (25). A number of studies have reported that probiotics can promote intestinal motility by modulating the gut microbiota to produce more short-chain fatty acids and regulate bile acid metabolism (20, 26, 27). Therefore, we hypothesized that specific probiotics may inhibit the intestinal absorption of Cd and increase the fecal excretion of Cd by the recovery of intestinal motility. This represents a new understanding of the mechanism underlying the protective effects of probiotics against Cd toxicity.

Based on these analyses, the aims of the present study were to perform a proteomic analysis to investigate the toxic mechanism of Cd on intestinal physiology. Experiments were also conducted to evaluate the protective effects of *L. plantarum* CCFM8610 against the Cd-induced depression of gut motility in mice.

## Materials and Methods

### Chemicals and Reagents

Fetal bovine serum (FBS), RPMI-1640 medium, trypsin, penicillin, and streptomycin were obtained from Gibco (Waltham, MA, USA). Cadmium chloride (CdCl_2_) and other analytical-grade laboratory reagents were obtained from Sinopharm Chemical Reagent Company (Shanghai, China). iTRAQ reagents were purchased from Applied Biosystems (Waltham, MA, USA).

Kits purchased from SenBeiJia Biotechnology (Nanjing, China) were used to determine the contents of nitric oxide (NO), 5-hydroxytryptamine (5-HT), substance P, acetylcholinesterase (AChE), vasoactive intestinal peptide (VIP), and calcitonin gene-related peptide (CGRP). TRIzol reagent was obtained from Life Technologies (Waltham, MA, USA). Reverse transcription was performed using a kit purchased from Takara Bio (Beijing, China).

### Cellular Experiment for Proteomic Analysis

HT-29 cells were conventionally incubated in RPMI-1640 medium containing 1% penicillin/streptomycin and 10% FBS. Cells were cultured at 37°C in a 5% CO_2_ moist atmosphere, and the medium was refreshed every 2 days. The cells were digested with trypsin and diluted to a 1:3 ratio for passage when the confluence reached 80–90%.

In the logarithmic growth phase, cells were grown in a six-well plate at a density of 1 × 10^6^ cells per well. After cell adhesion, the cells were divided into Control and Cd groups for treatment, as shown in **Table 1**. The dose of Cd exposure (50 μM CdCl_2_) was selected to mimic a moderate toxic effect of Cd on intestinal epithelial cells (28). After 24-h incubation, cells were collected for iTRAQ proteomic analysis by the method shown in **Supplemental Figure 1** (29). Protein Pilot Software V. 5.0 (AB Sciex, Beijing, China) was used for data processing. The identification of proteins and screening of differentially expressed proteins were performed as described in a previous study (29). The mass spectrometry proteomics data have been deposited to the ProteomeXchange Consortium (http://proteomecentral.proteomexchange.org) *via* the iProX partner repository (30) with the dataset identifier PXD022209.

**Table 1:** Cell experiment design.

Groups	Treatment
Control	1% penicillin/streptomycin +10% FBS +RPMI-1640
Cd	1% penicillin/streptomycin +10% FBS + RPMI-1640 + CdCl_2_ 50 μM)

### Strain Culture and Powder Manufacture

The incubation and lyophilization of *L. plantarum* CCFM8610 were performed as previously reported (21).

### Experimental Design

C57BL/6 mice (4 weeks of age) obtained from the Shanghai Laboratory Animal Center (Shanghai, China) were used in the experiments. The mice were kept in a temperature- and humidity-controlled room that was set to maintain a 12-h light/12-h dark cycle. The mice were fed standard commercial rat chow, and water was provided *ad libitum*. All the protocols of the study were approved by the Ethics Committee of Jiangnan University, China (JN. No. 20170618-20170918), and all the study procedures were performed in accordance with European Union guidelines (Directive 2010/63/EU) for the care and use of experimental animals.

After a 1-week adaptation period, 30 mice were divided into three groups, including the Control group, Cd group, and Cd plus CCFM8610 group (**Table 2**). The concentration of CdCl_2_ was selected based on the results of our previous study to mimic chronic oral Cd exposure (19). After a 10-week treatment, the mice were anesthetized and sacrificed. The blood, feces, and tissues of the mice were then collected for analysis.

**Table 2:** Animal experiment protocol.

Groups	Treatment
Control	SM + PW
Cd	SM + Cd
CCFM8610+Cd	SM + CCFM8610 + Cd

SM, 0.2 ml of skim milk once daily via gavage; PW, plain water for drinking; Cd, CdCl_2_ at 100 mg/L in drinking water; SM + CCFM8610, 1 × 10^9^ CFU L. plantarum CCFM8610 in 0.2 ml of skim milk once daily via gavage. The treatments lasted 10 weeks.

### Fecal Water Content

During the experiment, each mouse was housed in a pathogen-free cage and fecal samples were collected weekly. The fecal water content was determined using a method previously described (31).

### Determination of First Black Stool and Gastrointestinal (GI) Transit

The mice were fasted overnight and then gavaged with 0.2 ml of activated carbon solution. They were then given free access to food and water, and the time between the gavage and the appearance of their first darkened feces was recorded.

At the end of the experiment, each mouse was fasted overnight and the GI transit time was determined by recording the length of the small intestine and the distance traveled by the activated carbon in the intestine (32).

### Determination of Neurotransmitters in Tissues

The concentrations of NO, 5-HT, substance P, AchE, VIP, and CGRP in the colon and jejunum were determined using the kits, according to the manufacturer’s instructions.

### Determination of RT-qPCR

A quantitative reverse transcription polymerase chain reaction (RT-qPCR) was performed using a Bio-Rad CFX96TM Real-Time System (Shanghai, China), as previously reported (29). All the primers used in this test are listed in **Supplemental Table 1** (33). All assays were repeated three times, and the 2^-△△Ct^ method was used to analyze the data.

### Determination of Cd Levels in Tissues and Blood

The concentrations of Cd in the liver, kidneys, and blood were measured using a graphite furnace atomic absorption spectrophotometer (Varian, USA) (22).

### Statistical Analysis

The data are presented as means ± SD. A one-way analysis of variance was used for the intergroup analysis, with the differences considered to be statistically significant at *P* < 0.05. GraphPad Prism 8.0 (GraphPad Software Inc., San Diego, CA, USA) and OriginPro 8.5 (OriginLab Corporation, Northampton, MA, USA) were used for statistical analysis and construction.

## Results

### Cd-Induced Alterations in the Expression of Protein Profiles in HT-29 Cells

**Figure 1:**
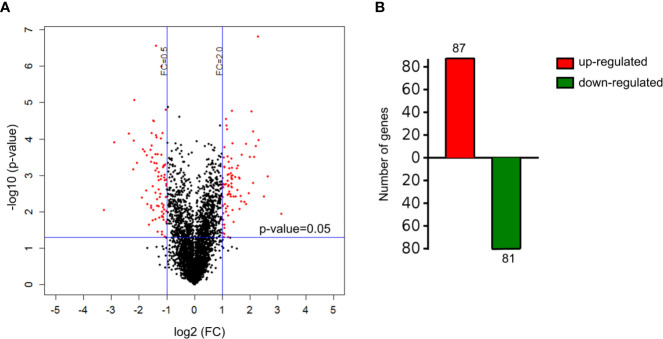
Differentially expressed proteins in HT-29 cells after Cd exposure. **(A)** volcano plots showing all detected proteins. The red dots represent the differentially expressed proteins after Cd exposure P-value of <0.05 with fold change of >2 or fold change of <0.5. The black dots are proteins with no significant changes. **(B)** the number of up-regulated and down-regulated genes responsible for differentially expressed proteins.

**Figure 2:**
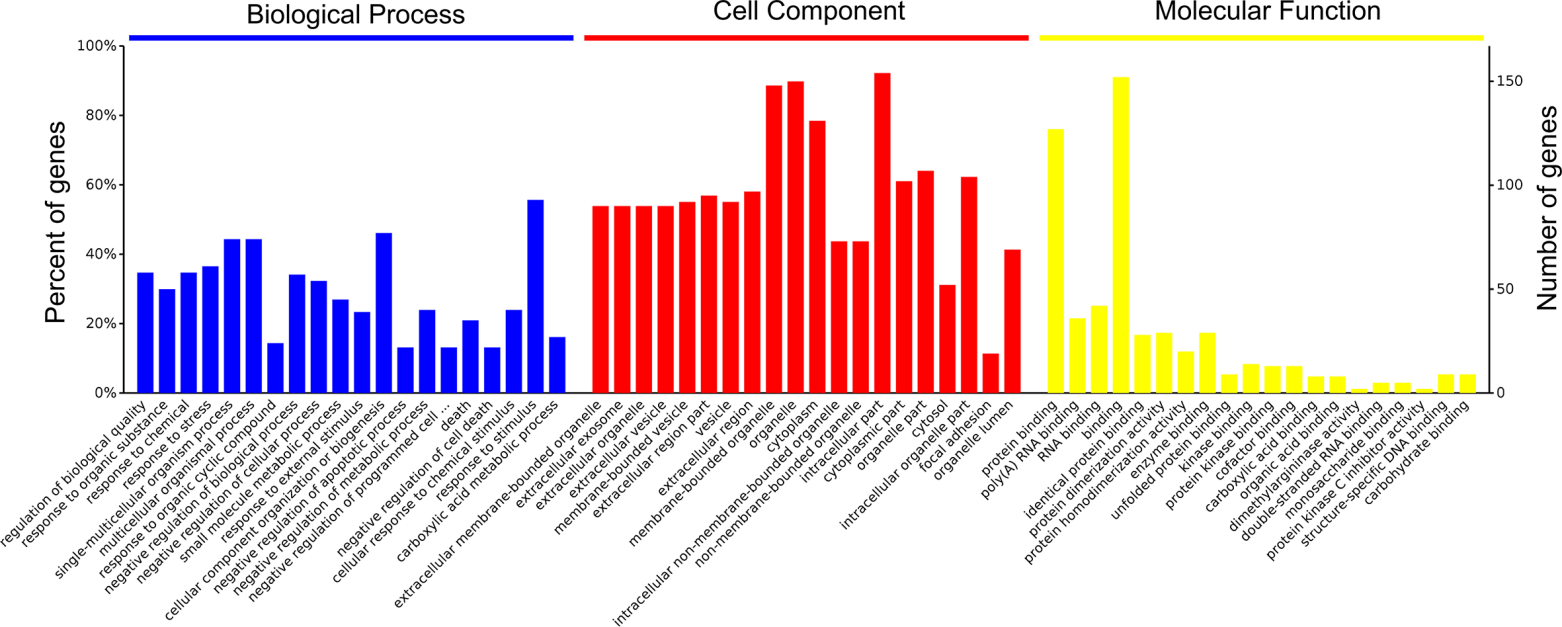
Functional categories of differentially expressed proteins after Cd-exposure by GO analysis.

A total of 4,089 proteins were detected and identified by iTRAQ analysis (**Figure 1A**). Based on the screening criteria of a fold change >2.0 and P value <0.05, Cd exposure was found to induce the differential expression of 168 proteins (**Figure 1B**). Gene ontology (GO) analysis (**Figure 2**) indicated that the differentially expressed proteins after Cd exposure had a wide range of functions that could be categorized as biological processes, cellular components, and molecular functions. The relevant metabolic pathways were related to stress response (19 proteins), membrane protein and other cell structure proteins (34 proteins), carbohydrate metabolism (13 proteins), lipid metabolism (10 proteins), amino acid metabolism (8 proteins), translation (8 proteins), etc. The Kyoto Encyclopedia of Genes and Genomes (KEGG) classification (**Supplemental Figure 2**) showed similar trends in the Cd-induced alterations in protein expression.

Of the proteins altered in the global stress response (**Supplemental Table 2**), eight heat-shock proteins were upregulated by a factor of 2–3 after Cd exposure. Cd also induced a 6.2-fold increase in the expression of a heme oxygenase protein HMOX1 (P09601). The expression of M6PR (P20645) protein, a mannose-6-phosphate receptor related to lysosomal transport and cell apoptosis, increased by a factor of 8.8 after Cd exposure. Some proteins responsible for protein synthesis and hydrolysis (e.g. TGM2, PSMG2, and PREP), the glycolytic pathway (e.g. ACSS1 and ALDOC), and lipid metabolism (e.g. PEBP1, CMBL, and PEBP1) were downregulated in the Cd-treated group. Other proteins associated with cellular ribosome synthesis (e.g., TCOF1 and GTPBP4) and the maintenance of cellular morphology (e.g., FLNB and FLNA) were upregulated in response to Cd stimulation.

### *L. plantarum* CCFM8610-Mediated Relief of the Cd-Induced Depression of Intestinal Motility

**Figure 3:**
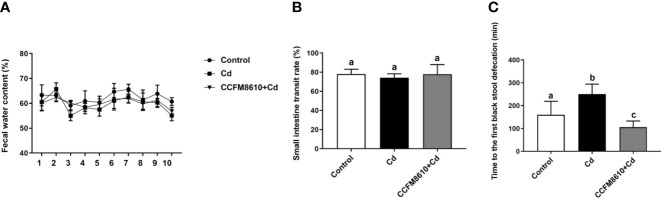
Effects of *L. plantarum* CCFM8610 on Cd-induced alterations in intestinal motility of mice. **(A)** Fecal water content of mice. **(B)** Small intestinal transit rate of mice. **(C)** First black stool defecation time of mice. The letters a, b and c mean the data of groups with different letters differ significantly (*P* < 0.05).

A chronic oral Cd exposure induced decreases in fecal water content and inhibited the GI transit ability of the mice (**Figure 3**). Treatment with *L. plantarum* CCFM8610 caused some recovery of the gut motility-related parameters of the mice, especially the time of the excretion of their first black feces (*P* < 0.05).

### *L. plantarum* CCFM8610-Mediated Recovery of the Cd-Induced Alterations in the Neurotransmitter Levels

**Figure 4:**
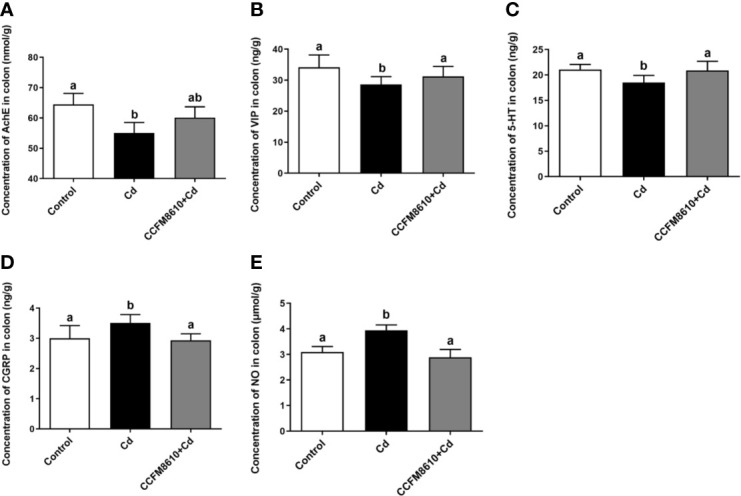
Effects of *L. plantarum* CCFM8610 on Cd-induced alterations in the levels of AchE **(A)**, VIP **(B)**, 5-HT **(C)**, CGRP **(D)**, and NO **(E)** in the colon of mice. The letters a and b mean the data of groups with different letters differ significantly *P* < 0.05). AchE, acetylcholinesterase; VIP, vasoactive intestinal peptide; 5-HT, 5-hydroxytryptamine; CGRP, calcitonin gene-related peptide; NO, nitric oxide.

The levels of the neurotransmitters AchE, VIP, and 5-HT in the colon were significantly reduced by Cd exposure, whereas the CGRP and NO levels were increased (*P* < 0.05, **Figure 4**). These alterations were reversed after the administration of *L. plantarum* CCFM8610. Similar protective effects on the levels of VIP and CGRP of this probiotic were observed in the jejunum (**Supplemental Figure 3**).

### *L. plantarum* CCFM8610-Mediated Protection Against Cd-Induced Cellular Stress in the Intestines

**Figure 5:**
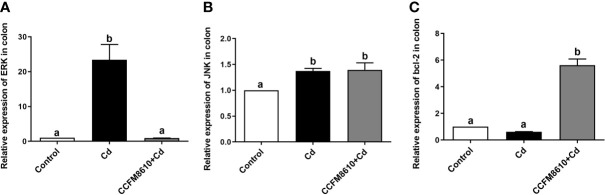
Effects of *L. plantarum* CCFM8610 on Cd-induced alterations in the expression of ERK **(A)**, JNK **(B)**, and bcl-2 **(C)** in the colon of mice. The letters a and b mean the data of groups with different letters differ significantly (*P* < 0.05). ERK, extracellular regulated protein kinases; JNK, c-Jun N-terminal kinase; Bcl-2, B-cell lymphoma-2.

Cd exposure induced significant extracellular regulated protein kinases (ERK) and c-Jun N-terminal kinase (JNK) expressions in the colon and jejunum of the mice (*P* < 0.05, **Figures 5A, B**, and **Supplemental Figures 4A, B**). The *L. plantarum* CCFM8610 treatment induced marked recovery of the alterations in these two genes, and increased the expression of B-cell lymphoma-2 (bcl-2) in the gut (**Figure 5C**, **Supplemental Figure 3**).

### *L. plantarum* CCFM8610-Mediated Reduction of Cd Levels in the Liver, Kidneys, and Blood of Mice

**Figure 6:**
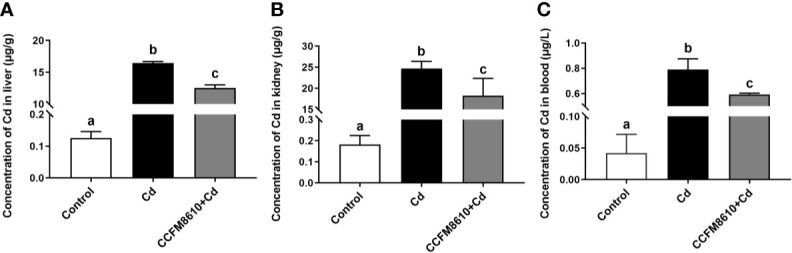
Effects of *L. plantarum* CCFM8610 on the concentrations of Cd in the liver **(A)**, kidney **(B)**, and blood **(C)** of mice after Cd exposure. The letters a, b and c mean the data of groups with different letters differ significantly (*P* < 0.05).

Compared with the control group, Cd exposure caused significant increases in the Cd levels in the tissues and blood of the mice (*P* < 0.05, **Figure 6**). The *L. plantarum* CCFM8610 treatment led to a marked reduction in the levels of Cd in the blood, liver, and kidneys of the mice (**Figure 6**).

## Discussion

The toxicity of Cd in the gut is attracting increasing attention because food-borne Cd is the main source of exposure to this environmental hazard (15). Besides, iTRAQ is a widely used approach for proteomic analysis that identifies and quantifies proteins using labeled peptides identifiable by sensitive mass spectrometers (34). This analysis is further strengthened by using robust bioinformatic tools and statistical analyses to support the observations (35). Some reviews have summarized the application of proteomic analysis on the studies of the toxicity of heavy metals including lead, arsenic and Cd (36, 37). Using the proteomic techniques is helpful for the screening of highly sensitive and specific biomarkers of metal exposure. To understand the molecular toxicity mechanism of Cd on enterocytes, alterations in the proteomic profiles after Cd exposure were evaluated (**Figures 1**, **2**, and **Supplemental Table 2**). The results of iTRAQ analysis revealed that Cd treatment affected the expression of proteins involved in the stress response and cell apoptosis in enterocytes, which is consistent with previous findings regarding the cytotoxic effects of Cd exposure (38, 39). In support of these results, our animal trial showed that oral Cd exposure induced a significant toxic effect on intestinal physiology (**Figure 3**–**6**, **Supplemental Figure 3**, **4**). Based on previous studies, heavy metals including copper and mercury can adversely affect gut physiology *via* inducing oxidative stress and inflammation and disrupting the metabolic homeostasis of the gut microbiota (40, 41). Mycotoxins such as deoxynivalenol, aflatoxin and ochratoxin have been reported to inhibit the production of intestinal mucins and damage the gut barrier (14, 42, 43). Some of these studies reported that the effects on MAPK pathway may be an important mechanism for the toxicity of these hazards.

Abnormal increase in the expression of heat-shock proteins may be accompanied by enhanced cell proliferation and increased tumor risk (44). The induction of heme oxygenase expression, which is reported to be beneficial against inflammation and tissue injury, may be a self-protective mechanism of the enterocytes against Cd exposure (45). Previous studies have indicated that NO can induce the expression of heme oxygenase-1 *via* activation of the ERK pathway (46, 47). This finding was confirmed by our *in vivo* results, which showed that Cd exposure increased the level of NO and the expression of ERK in the gut of mice (**Figures 4** and **5**). The most drastic changes in expression were observed in the cation-dependent mannose-6-phosphate receptor (increase by 8.8-fold), a protein associated with lysosomal transport and cell apoptosis (48), which indicates a strong Cd-induced stress response in enterocytes.

Among other proteins significantly affected by Cd exposure, the aldo-keto reductase family 1 member B10 has been reported to be involved in the CRAF-MEK-ERK pathway (49), which is consistent with the results of our animal experiment (**Figure 5**). Some proteins related to amino acid metabolism, such as N(G),N(G)-dimethylarginine dimethylaminohydrolase 1 and 2, were downregulated by the Cd exposure (**Supplemental Table 2**). This can explain the increase in the intestinal NO levels in the gut of Cd-exposed mice, as these enzymes play a role in reducing the production and bioavailability of NO (50).

Previous studies have shown that specific probiotics can provide significant protective effects against Cd toxicity *in vivo* (17, 22). The reported mechanisms include intestinal Cd sequestration, anti-oxidative stress action, and bile acid modulation (19, 21). In the present study, we also noticed that the administration of *L. plantarum* CCFM8610 alleviated Cd-induced intestinal motility disorders (**Figure 3A**). Such protective effects may increase the fecal excretion of Cd and inhibit the absorption of this metal in the gut (11). As such, it may represent an additional mechanism by which this strain reduces the Cd burden in the tissues and blood of mice (**Figure 6**). Previous studies have revealed the potential mechanism for the modulation of neurotransmitters by specific gut microbes. Gut microbiota promote colonic 5-HT production through an effect of short-chain fatty acids on enterochromaffin cells (51). Moreover, secondary bile acids produced by microbial biotransformation of cholate can stimulate neurotransmitters biosynthesis (52). Some lactobacillus strains such as *L. reuteri* and *L. rhamnosus* have been reported to activate GABA and 5-HT receptors in the gut, which can also explain the effects of probiotics on the levels of neurotransmitters (53, 54). In support of the proteomic analysis results, Cd exposure was also observed to increase the NO level in the intestines of mice (**Figure 4E**, **Supplemental Figure 3E**). This nitroso compound is reported to serve as an inhibitory transmitter in the gut of humans and animals (55, 56). Clinical studies have shown that the levels of NO and CGRP (a neuropeptide affecting a variety of gastrointestinal functions) are increased in patients with constipation (57, 58). The recovery of these two neurotransmitters by *L. plantarum* CCFM8610 treatment may therefore relieve the inhibitory effects of Cd on intestinal motility.

Substance P, AchE, VIP, and 5-HP play important roles in stimulating intestinal motility, relaxing enteric smooth muscle, and promoting defecation (59). Previous studies have reported decreased levels of these neurotransmitters in patients with idiopathic chronic constipation and constipation-predominant irritable bowel syndrome (60–62). The protective effects of *L. plantarum* CCFM8610 against Cd-induced intestinal motility disorders may also be due to the strain’s ability to increase the levels of these parameters. In support of our hypothesis, previous studies have shown that other probiotics, including *L. plantarum* 90sk and *L. casei* Qian, can modulate the expression of these neurotransmitters in rodents and humans (63–65).

Cd exposure has been reported to induce oxidative stress, which activates the mitogen-activated protein kinase (MAPK) signaling pathway and results in programmed cell death (66). This finding is consistent with our proteomic analysis results, as we found that proteins related to MAPK pathways (such as heme oxygenase-1 and heat shock protein-70) increased after Cd treatment. As shown in **Figures 5A, B**, and **Supplemental Figures 4A, B**, the expressions of protein kinases ERK and JNK were significantly increased after Cd exposure, further confirming the cytotoxic effects of Cd on enterocytes. Bcl-2 is known to be a key regulator of cell apoptosis and death (67). A previous study reported that the expression of bcl-2 can suppress Cd-induced cell death (68). *L. plantarum* CCFM8610 treatment induced the recovery of Cd-induced alterations in the expression of ERK and bcl-2, which indicates that this strain may inhibit Cd-activated programmed cell death and thereby protect intestinal neuronal cells. This finding may also explain the *L. plantarum* CCFM8610-mediated alleviation of the Cd-induced depression of intestinal motility. Consistent with our results, previous studies have shown that probiotics such as *L. reuteri* 6475 can suppress the ERK1/2 pathway in tumor necrosis factor-treated human myeloid leukemia-derived cells (69), and that a multispecies probiotic product increased the bcl-2 levels in constipated mice (70).

In this study, iTRAQ analyses were performed to evaluate the toxic effects of Cd on enterocytes. The results revealed that changes in the proteomic profiles after Cd exposure were related to pathways involved in the stress response and carbohydrate metabolism. The results of animal trials indicated that *L. plantarum* CCFM8610 treatment provided protective effects against dysfunctions of Cd-induced intestinal motility *via* the recovery of neurotransmitter levels and suppression of the cellular stress response in mice.

## Data Availability Statement

The datasets presented in this study can be found in online repositories. The names of the repository/repositories and accession number(s) can be found in the article/**Supplementary Material**.

## Ethics Statement

The animal study was reviewed and approved by the Ethics Committee of Jiangnan University, China (JN. No. 20170618-20170918).

## Author Contributions

YL: Methodology, Software, Formal analysis, Visualization, Writing—original draft. JW: Writing—original draft, Methodology, Software. YX: Investigation, Formal analysis. QL: Software, Formal analysis. LY: Validation, Methodology, Investigation. FT: Validation, Investigation. JZ: Validation, Investigation. HZ: Validation, Investigation. QZ: Conceptualization, Writing—review and editing, Supervision, Funding acquisition. WC: Project administration, Funding acquisition. All authors contributed to the article and approved the submitted version.

## Funding

This work was supported by the National Natural Science Foundation of China Program (No. 31871773, 32001665 and No. 31820103010); National First-Class Discipline Program of Food Science and Technology (JUFSTR20180102); the BBSRC Newton Fund Joint Centre Award; and Collaborative Innovation Center of Food Safety and Quality Control in Jiangsu Province.

## Conflict of Interest

The authors declare that the research was conducted in the absence of any commercial or financial relationships that could be construed as a potential conflict of interest.
