# Supplementary figures and images for: Relief of Cadmium-Induced Intestinal Motility Disorder in Mice by Lactobacillus plantarum CCFM8610
Source: Front Immunol. 2020 Dec 10;11:619574. doi: 10.3389/fimmu.2020.619574 (PMC7758470; doi:10.3389/fimmu.2020.619574)

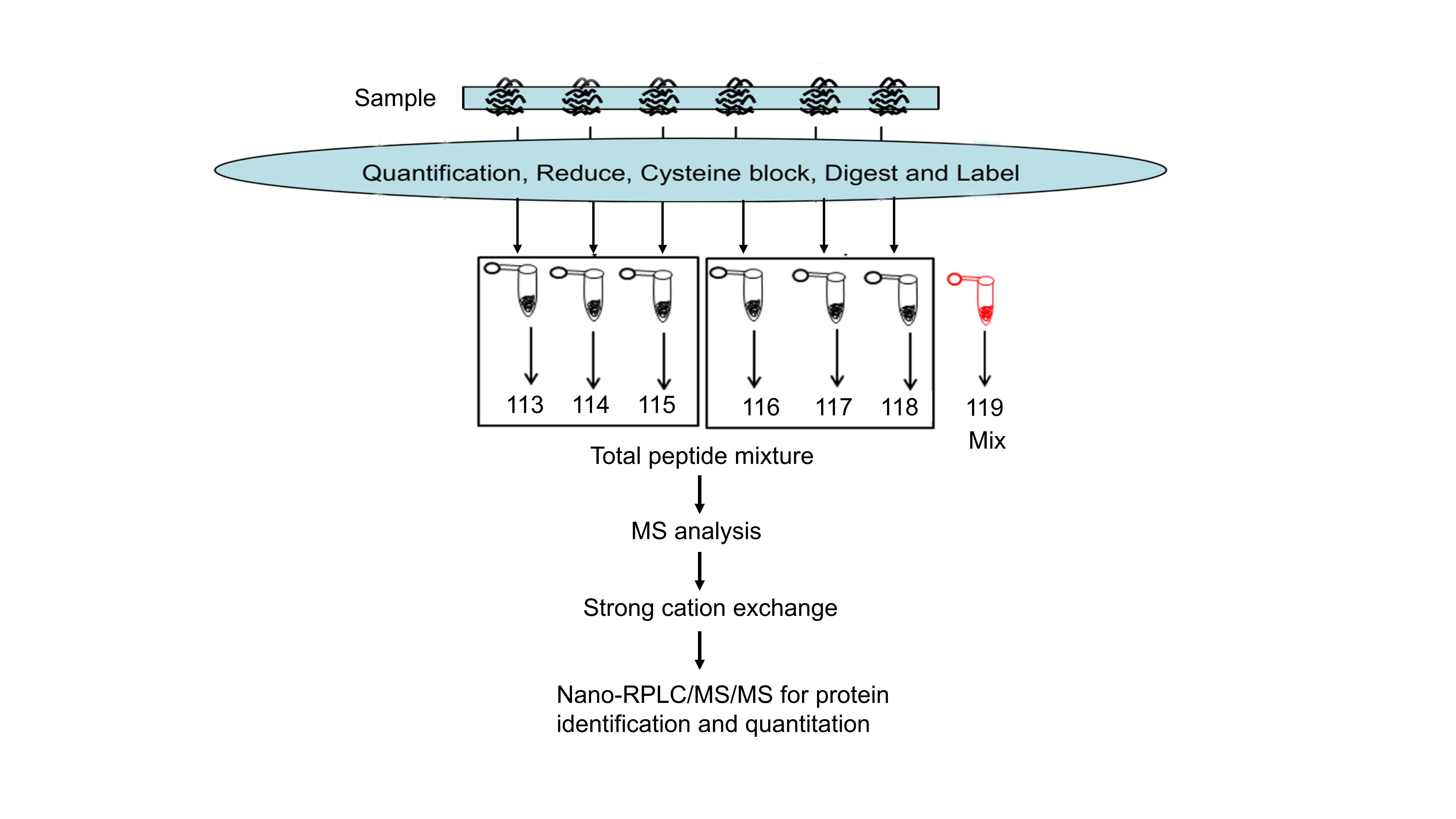

Supplement: Supplementary file 1 [file Image_1.tif]

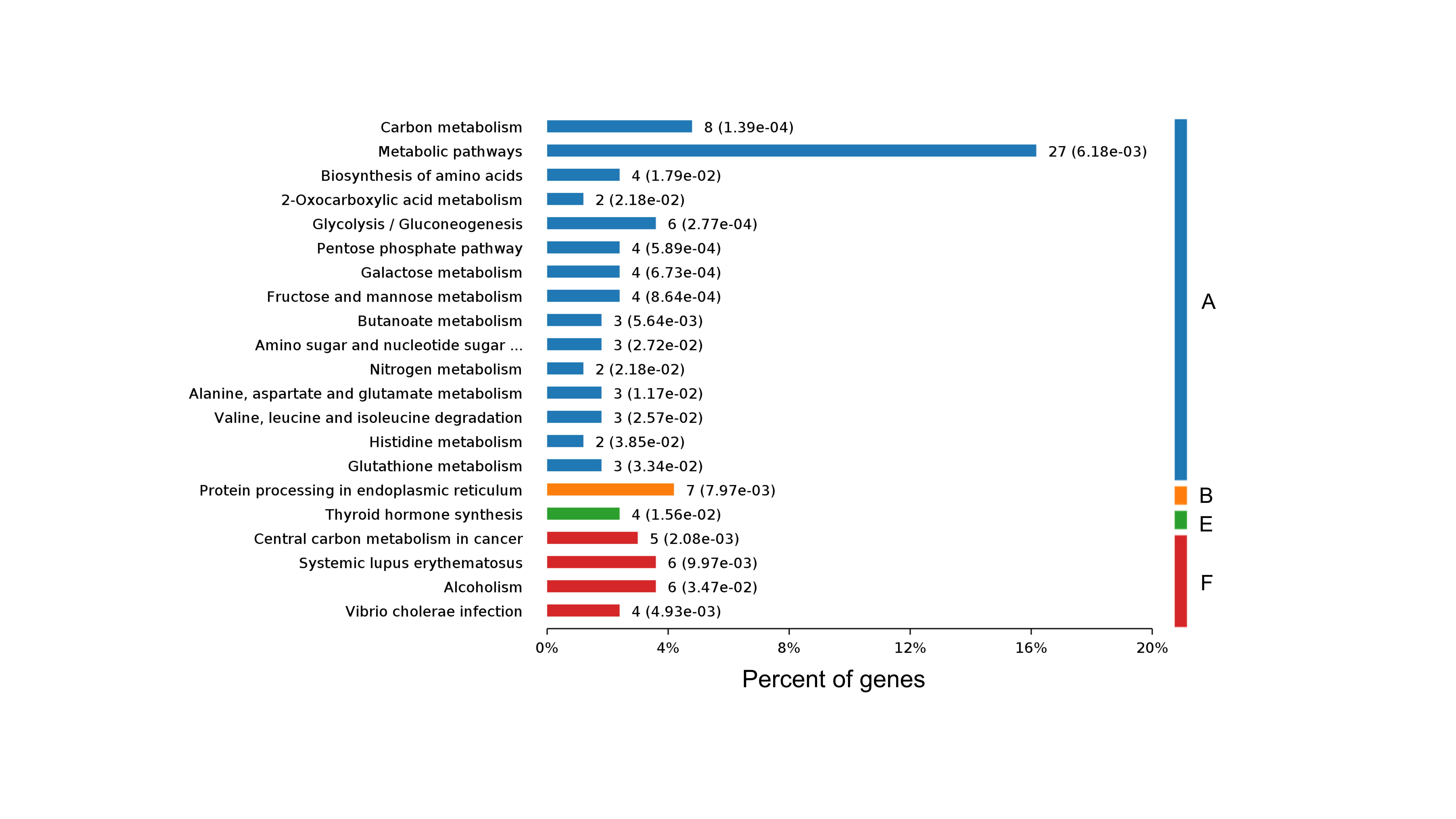

Supplement: Supplementary file 2 [file Image_2.tif]

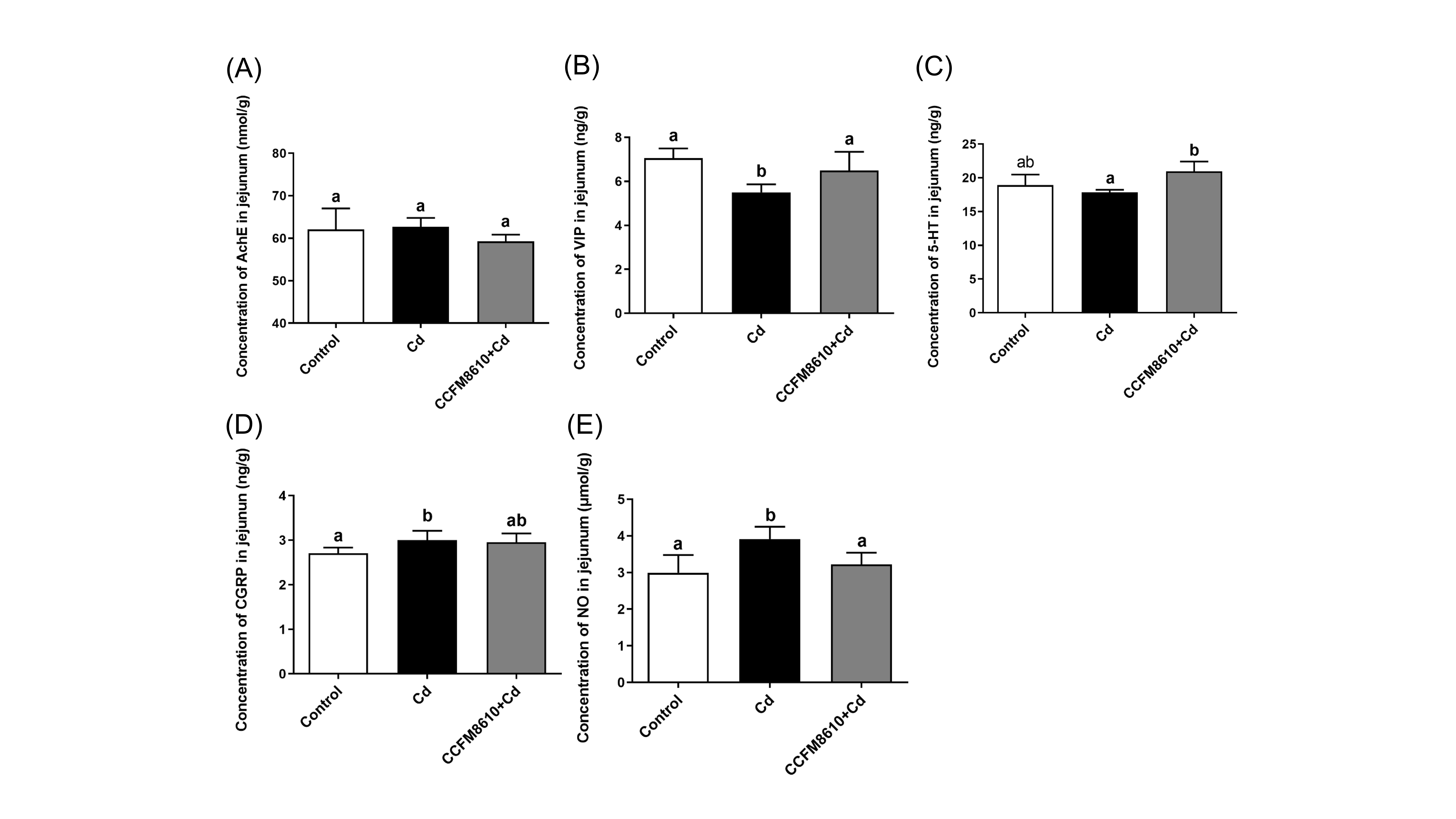

Supplement: Supplementary file 3 [file Image_3.tif]

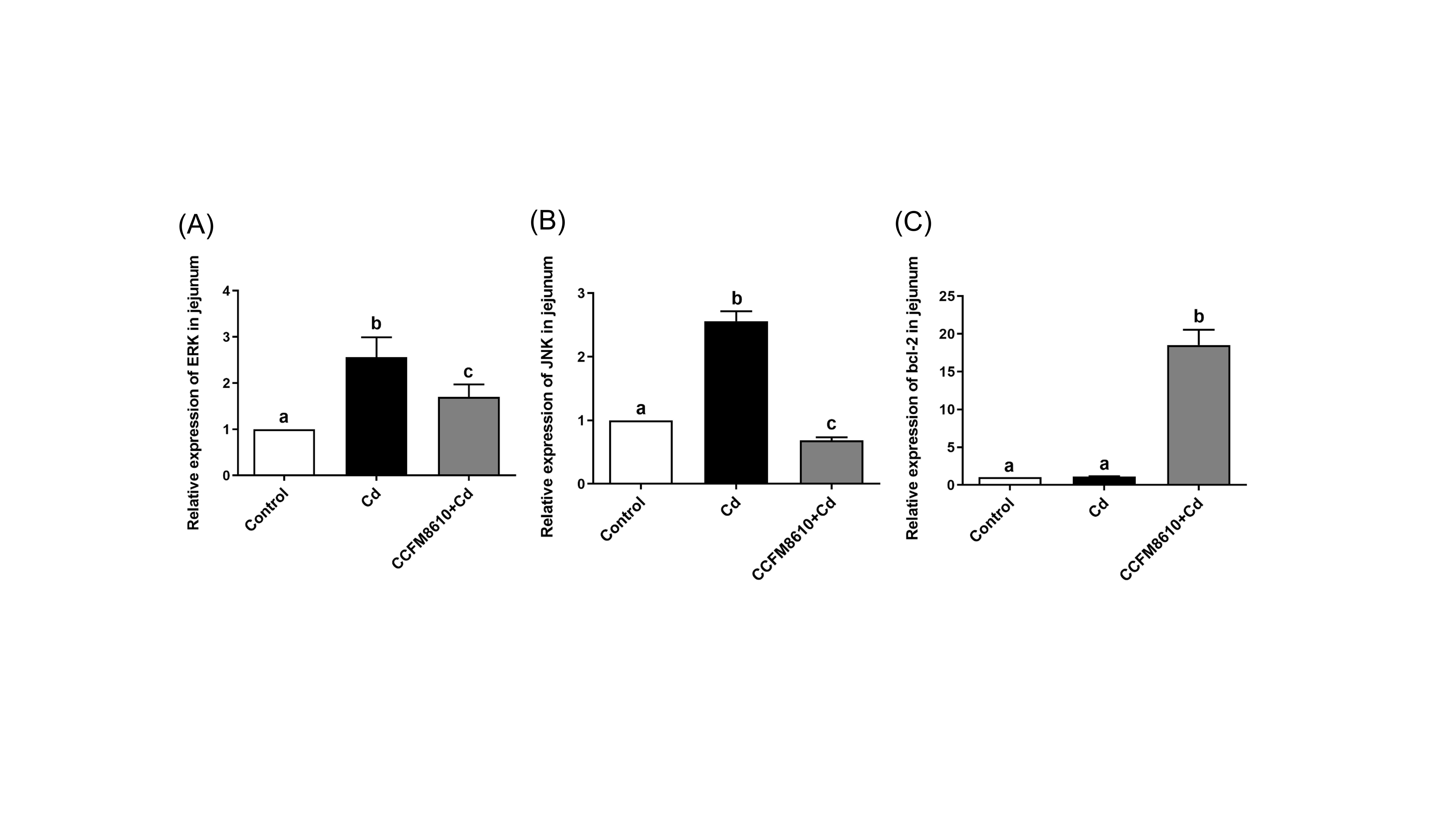

Supplement: Supplementary file 4 [file Image_4.tif]
