# Supplementary material for: Relief of Cadmium-Induced Intestinal Motility Disorder in Mice by Lactobacillus plantarum CCFM8610
Source: Front Immunol. 2020 Dec 10;11:619574. doi: 10.3389/fimmu.2020.619574 (PMC7758470; doi:10.3389/fimmu.2020.619574)
Supplement: Supplementary file 5 [file Table_1.docx]

Supplementary Table 1 Primer sequences for qRT-PCR

| Primer | Sequences |
| --- | --- |
| BCL2 F | 5’-GTGGCCTTCTTTGAGTTCG-3’ |
| BCL2 R | 5’-TCCCAGCCTCCGTTATCC-3’ |
| JNK1 F | 5’-CCAGCACCCATACATCAA-3’ |
| JNK1 R | 5’-GACAGACGGCGAAGACGA-3’ |
| ERK F | 5’-AGGATGTTAGGCTTCGTC-3 |
| ERK R | 5’-AAGCTATTCCTTCCCATG-3 |
| Gapdh F | 5’-GAGGGACTGCCTGGTGT-3’ |
| Gapdh R | 5’-GAGGGACTGCCTGGTGT-3’ |

F, forward primer; R, reverse primer.
