# Supplementary material for: Relief of Cadmium-Induced Intestinal Motility Disorder in Mice by Lactobacillus plantarum CCFM8610
Source: Front Immunol. 2020 Dec 10;11:619574. doi: 10.3389/fimmu.2020.619574 (PMC7758470; doi:10.3389/fimmu.2020.619574)
Supplement: Supplementary file 6 [file Table_2.docx]

Supplementary Table 2 Differentially expressed proteins in HT-29 cells after Cd exposure.

| Classification ^a^ | Accession ^b^ | Description ^c^ | FC ^d^ |
| --- | --- | --- | --- |
| Carbohydrate metabolism | Q9ULU4 | ZMYND8, Protein kinase C-binding protein 1 | -0.5 |
|  | P11216 | PYGB, Glycogen phosphorylase, brain form | -0.36 |
|  | P11413 | G6PD, Glucose-6-phosphate 1-dehydrogenase | +2.28 |
|  | O75874 | IDH1, Isocitrate dehydrogenase (NADP) cytoplasmic | -0.4 |
|  | P36871 | PGM1, Phosphoglucomutase-1 | -0.31 |
|  | Q9NUB1 | ACSS1, Acetyl-coenzyme A synthetase 2-like, mitochondrial | -0.48 |
|  | P09467 | FBP1, Fructose-1, 6-bisphosphatase 1 | -0.22 |
|  | Q14376 | GALE, UDP-glucose 4-epimerase | -0.4 |
|  | P09972 | ALDOC, Fructose-bisphosphate aldolase C | -0.46 |
|  | Q96C23 | GALM, Galactose mutarotase | -0.49 |
|  | O00462 | MANBA, Beta-mannosidase | -0.47 |
|  | P34949 | MPI, Mannose-6-phosphate isomerase | -0.5 |
|  | O60218 | AKR1B10, Aldo-keto reductase family 1 member B10 | +4.96 |
| Amino acid metabolism | Q96GA7 | SDSL, Serine dehydratase-like | -0.47 |
|  | Q92876 | KLK6, Kallikrein-6 | +2.01 |
|  | P08243 | ASNS, Asparagine synthetase [glutamine-hydrolyzing] | +3.1 |
|  | P21964 | COMT, Catechol O-methyltransferase | -0.39 |
|  | P50135 | HNMT, Histamine N-methyltransferase | -0.41 |
|  | P51649 | ALDH5A1, Succinate-semialdehyde dehydrogenase, mitochondrial | -0.39 |
|  | O94760 | DDAH1, N(G), N(G)-dimethylarginine dimethylaminohydrolase 1 | -0.47 |
|  | O95865 | DDAH2, N(G), N(G)-dimethylarginine dimethylaminohydrolase 2 | -0.44 |
| Protein synthesis and hydrolysis | O00469 | PLOD2, Procollagen-lysine, 2-oxoglutarate 5-dioxygenase 2 | -0.46 |
|  | P21980 | TGM2, Protein-glutamine gamma-glutamyltransferase 2 | -0.34 |
|  | Q969U7 | PSMG2, Proteasome assembly chaperone 2 | -0.44 |
|  | P48147 | PREP, Prolyl endopeptidase | -0.5 |
|  | P07339 | CTSD, Cathepsin D | +2.35 |
|  | P12955 | PEPD, Xaa-Pro dipeptidase | -0.43 |
|  | Q03405 | PLAUR, Urokinase plasminogen activator surface receptor | +2.28 |
|  | Q9H4A4 | RNPEP, Aminopeptidase B | -0.5 |
|  | P27487 | DPP4, Dipeptidyl peptidase 4 | -0.43 |
|  | Q9Y5A7 | NUB1, NEDD8 ultimate buster 1 | -0.44 |
|  | O43291 | SPINT2, Kunitz-type protease inhibitor 2 | +2.32 |
| Nucleic acid metabolism | Q9NR30 | DDX21, Nucleolar RNA helicase 2 | +4.19 |
|  | Q9NVP1 | DDX18, ATP-dependent RNA helicase DDX18 | +2.55 |
|  | P13489 | RNH1, Ribonuclease inhibitor | -0.4 |
|  | Q14498 | RBM39, RNA-binding protein 39 | +2.03 |
|  | Q8IY81 | FTSJ3, pre-rRNA 2'-O-ribose RNA methyltransferase FTSJ3 | +2.13 |
|  | Q14240 | EIF4A2, Eukaryotic initiation factor 4A-II | -0.35 |
|  | Q14197 | MRPL58, Peptidyl-tRNA hydrolase ICT1, mitochondrial | +2.57 |
| Lipid metabolism | P54868 | HMGCS2, Hydroxymethylglutaryl-CoA synthase, mitochondrial | -0.24 |
|  | P52895 | AKR1C2, Aldo-keto reductase family 1 member C2 | +3.11 |
|  | Q9H0W9 | C11orf54, Ester hydrolase C11orf54 | -0.36 |
|  | Q96DG6 | CMBL, Carboxymethylenebutenolidase homolog | -0.38 |
|  | P30086 | PEBP1, Phosphatidylethanolamine-binding protein 1 | -0.49 |
|  | Q8IY17 | PNPLA6, Patatin-like phospholipase domain-containing protein 6 | -0.32 |
|  | P51648 | ALDH3A2, Aldehyde dehydrogenase family 3 member A2 | +2.28 |
|  | P16219 | ACADS, Short-chain specific acyl-CoA dehydrogenase, mitochondrial | -0.22 |
|  | P07108 | DBI, Acyl-CoA-binding protein | -0.38 |
|  | Q5W0Z9 | ZDHHC20, Palmitoyltransferase ZDHHC20 | +3.64 |
| Cell morphology maintenance | Q9BW30 | TPPP3, Tubulin polymerization-promoting protein family member 3 | -0.19 |
|  | P06396 | GSN, Gelsolin | -0.22 |
|  | Q15149 | PLEC, Plectin | +2.28 |
|  | P35579 | MYH9, Myosin-9 | +2.92 |
|  | O75369 | FLNB, Filamin-B | +2.13 |
|  | P21333 | FLNA, Filamin-A | +3.98 |
|  | P08727 | KRT19, Keratin, type I cytoskeletal 19 | +2.02 |
|  | P06753 | TPM3, Tropomyosin alpha-3 chain | +2.5 |
|  | Q14019 | COTL1, Coactosin-like protein | +2.78 |
|  | Q9NYT0 | PLEK2, Pleckstrin-2 | -0.45 |
|  | Q5SW79 | CEP170, Centrosomal protein of 170 kDa | +3.47 |
| Transcription translation | P11387 | TOP1, DNA topoisomerase 1 | +3.28 |
|  | Q32MZ4 | LRRFIP1, Leucine-rich repeat flightless-interacting protein 1 | +2.11 |
|  | Q9H307 | PNN, Pinin | +2.31 |
|  | P67809 | YBX1, Y-box-binding protein 1 | +2.13 |
|  | P40763 | STAT3, Signal transducer and activator of transcription 3 | -0.47 |
|  | Q96DH6 | MSI2, RNA-binding protein Musashi homolog 2 | -0.41 |
|  | Q9BTE6 | AARSD1, Alanyl-tRNA editing protein Aarsd1 | -0.38 |
|  | Q9UBP6 | METTL1, tRNA (guanine-N (7) -)-methyltransferase | -0.37 |
| Transporter | P20591 | MX1, Interferon-induced GTP-binding protein Mx1 | -0.41 |
|  | P42704 | LRPPRC, Leucine-rich PPR motif-containing protein, mitochondrial | +2.21 |
|  | Q9Y3Y2 | CHTOP, Chromatin target of PRMT1 protein | +2.34 |
|  | P08195 | SLC3A2, 4F2 cell-surface antigen heavy chain | +4.62 |
|  | Q15758 | SLC1A5, Neutral amino acid transporter B (0) | +2.58 |
|  | P30825 | SLC7A1, High affinity cationic amino acid transporter 1 | +2.95 |
|  | Q01650 | SLC7A5, Large neutral amino acids transporter small subunit 1 | +4.48 |
|  | Q9UNH7 | SNX6, Sorting nexin-6 | -0.46 |
|  | Q9HAV7 | GRPEL1, GrpE protein homolog 1, mitochondrial | +2.81 |
|  | P04920 | SLC4A2, Anion exchange protein 2 | +2.12 |
|  | P55011 | SLC12A2, Solute carrier family 12 member 2 | -0.35 |
|  | Q16555 | DPYSL2, Dihydropyrimidinase-related protein 2 | -0.48 |
|  | P04083 | ANXA1, Annexin A1 | +2.38 |
|  | P08758 | ANXA5, Annexin A5 | -0.44 |
|  | Q9HD45 | TM9SF3, Transmembrane 9 superfamily member 3 | +2.02 |
|  | Q9UI12 | ATP6V1H, V-type proton ATPase subunit H | -0.48 |
|  | Q9H0T7 | RAB17, Ras-related protein Rab-17 | -0.34 |
| cell apoptosis | Q13501 | SQSTM1, Sequestosome-1 | +4.93 |
|  | P20645 | M6PR, Cation-dependent mannose-6-phosphate receptor | +8.8 |
|  | P61626 | LYZ, Lysozyme C | -0.48 |
|  | O75143 | ATG13, Autophagy-related protein 13 | -0.4 |
|  | P09327 | VIL1, Villin-1 | -0.44 |
|  | Q8TAX9 | GSDMB, Gasdermin-B | -0.42 |
|  | P29590 | PML, Protein PML | -0.34 |
| Global Stress Response | Q14451 | GRB7, Growth factor receptor-bound protein 7 | -0.39 |
|  | O76070 | SNCG, Gamma-synuclein | -0.35 |
|  | Q8TD30 | GPT2, Alanine aminotransferase 2 | +2.23 |
|  | P35754 | GLRX, Glutaredoxin-1 | -0.27 |
|  | P18283 | GPX2, Glutathione peroxidase 2 | -0.45 |
|  | P13667 | PDIA4, Protein disulfide-isomerase A4 | +2.58 |
|  | P09601 | HMOX1, Heme oxygenase 1 | +6.23 |
|  | P11166 | SLC2A1, Solute carrier family 2, facilitated glucose transporter member 1 | +4.36 |
|  | O76003 | GLRX3, Glutaredoxin-3 | +2.3 |
|  | Q9UHD1 | CHORDC1, Cysteine and histidine-rich domain-containing protein 1 | +2.29 |
|  | P14625 | HSP90B1, Endoplasmin | +2.52 |
|  | Q9UBS4 | DNAJB11, DnaJ homolog subfamily B member 11 | +2.05 |
|  | P11021 | HSPA5, Endoplasmic reticulum chaperone BiP | +2.51 |
|  | P08238 | HSP90AB1, Heat shock protein HSP 90-beta | +2.38 |
|  | P10809 | HSPD1, 60 kDa heat shock protein, mitochondrial | +2.05 |
|  | Q92598 | HSPH1, Heat shock protein 105 kDa | +2.08 |
|  | P38646 | HSPA9, Stress-70 protein, mitochondrial | +2.05 |
|  | P04792 | HSPB1, Heat shock protein beta-1 | -0.36 |
|  | P17066 | HSPA6, Heat shock 70 kDa protein 6 | +3.19 |
| Vitamin metabolism | P13716 | ALAD, Delta-aminolevulinic acid dehydratase | -0.47 |
|  | P13995 | MTHFD2, Bifunctional methylenetetrahydrofolate dehydrogenase/cyclohydrolase, mitochondrial | +2.03 |
|  | P29373 | CRABP2, Cellular retinoic acid-binding protein 2 | -0.38 |
| electron transport | P35270 | SPR, Sepiapterin reductase | -0.39 |
|  | P43490 | NAMPT, Nicotinamide phosphoribosyltransferase | +2.01 |
|  | P49821 | NDUFV1, NADH dehydrogenase (ubiquinone) flavoprotein 1, mitochondrial | +2.04 |
| nitrogen metabolism | P00918 | CA2, Carbonic anhydrase 2 | -0.33 |
|  | Q16790 | CA9, Carbonic anhydrase 9 | -0.44 |
| Cellular chromatin | O60264 | SMARCA5, SWI/SNF-related matrix-associated actin-dependent regulator of chromatin subfamily A member 5 | +2.22 |
|  | Q99877 | H2BC15, Histone H2B type 1-N | +3.03 |
|  | P62805 | H4C1, Histone H4 | +2.11 |
|  | Q71DI3 | H3C15, Histone H3.2 | +2.35 |
|  | P16401 | H1-5, Histone H1.5 | +2.2 |
|  | P07305 | H1-0, Histone H1.0 | -0.5 |
|  | Q9NRL2 | BAZ1A, Bromodomain adjacent to zinc finger domain protein 1A | +2.08 |
|  | P05204 | HMGN2, Non-histone chromosomal protein HMG-17 | +2.87 |
|  | Q71UI9 | H2AFV, Histone H2A.V | +3.89 |
|  | Q16778 | H2BC21, Histone H2B type 2-E | +2.21 |
|  | P68431 | H3C1, Histone H3.1 | +2.65 |
|  | P16403 | HIST1H1C, Histone H1.2 | +2.46 |
|  | P17096 | HMGA1, High mobility group protein HMG-I/HMG-Y | +2.36 |
| cell multiplication | Q9HCY8 | S100A14, Protein S100-A14 | +2.35 |
|  | P31947 | SFN, 14-3-3 protein sigma | -0.31 |
|  | Q04695 | KRT17, Keratin, type I cytoskeletal 17 | +2.5 |
|  | Q9UKY7 | CDV3, Protein CDV3 homolog | +2.06 |
|  | Q7Z3K3 | POGZ, Pogo transposable element with ZNF domain | -0.4 |
|  | P29317 | EPHA2, Ephrin type-A receptor 2 | +2.54 |
|  | Q9Y624 | F11R, Junctional adhesion molecule A | +2.05 |
|  | P04004 | VTN, Vitronectin | +2.51 |
|  | P56470 | LGALS4, Galectin-4 | -0.29 |
| cell nucleus | O15213 | WDR46, WD repeat-containing protein 46 | +2.64 |
|  | Q96HA1 | POM121, Nuclear envelope pore membrane protein POM 121 | +2.53 |
|  | Q13428 | TCOF1, Treacle protein | +2.11 |
|  | Q9BZE4 | GTPBP4, Nucleolar GTP-binding protein 1 | +2.61 |
|  | Q9NX58 | LYAR, Cell growth-regulating nucleolar protein | +2.57 |
|  | Q13601 | KRR1, KRR1 small subunit processome component homolog | +2.03 |
|  | P19338 | NCL, Nucleolin | +2.51 |
|  | P06748 | NPM1, Nucleophosmin | +2.54 |
| others | Q9Y6R7 | FCGBP, IgGFc-binding protein | -0.28 |
|  | O60264 | SMARCA5, SWI/SNF-related matrix-associated actin-dependent regulator of chromatin subfamily A member 5 | +2.22 |
|  | Q13228 | SELENBP1, Methanethiol oxidase | -0.14 |
|  | P06703 | S100A6, Protein S100-A6 | -0.1 |
|  | P48200 | IREB2, Iron-responsive element-binding protein 2 | -0.45 |
|  | A6NDB9 | PALM3, Paralemmin-3 | -0.41 |
|  | P06454 | PTMA, Prothymosin alpha | +2.11 |
|  | P01833 | PIGR, Polymeric immunoglobulin receptor | -0.38 |
|  | P30085 | CMPK1, UMP-CMP kinase | -0.42 |
|  | Q12797 | ASPH, Aspartyl/asparaginyl beta-hydroxylase | +3.64 |
|  | Q9P1F3 | ABRACL, Costars family protein ABRACL | -0.46 |
|  | P30039 | PBLD, Phenazine biosynthesis-like domain-containing protein | -0.3 |
|  | Q8IWE2 | FAM114A1, Protein NOXP20 | -0.36 |
|  | P02768 | ALB, Albumin | +5.71 |
|  | Q6NXE6 | ARMC6, Armadillo repeat-containing protein 6 | -0.44 |
|  | Q14914 | PTGR1, Prostaglandin reductase 1 | -0.28 |
|  | Q8TD06 | AGR3, Anterior gradient protein 3 | -0.46 |
|  | P43487 | RANBP1, Ran-specific GTPase-activating protein | +3.21 |
|  | P15408 | FOSL2, Fos-related antigen 2 | +4.36 |

^a^ Classification of differentially expressed proteins according to their functions annotated in the database of Uniprot and KEGG. ^b^ Accession number of each protein in the Uniprot database. ^c^ Description of each differentially expressed protein, including corresponding gene name of each protein and full protein name. ^d^ FC indicates fold change of each differently expressed protein in HT-29 cells after Cd exposure. Positive values indicate up-regulation of proteins and negative values indicate down-regulation.
