# Supplementary material for: Relief of Cadmium-Induced Intestinal Motility Disorder in Mice by Lactobacillus plantarum CCFM8610
Source: Front Immunol. 2020 Dec 10;11:619574. doi: 10.3389/fimmu.2020.619574 (PMC7758470; doi:10.3389/fimmu.2020.619574)
Supplement: Supplementary file 7 [file Table_3.docx]

Fig. S1. workflow of iTRAQ analysis. Each sample was labeled with one of eight iTRAQ reagents. The three peptide samples from the control group were respectively labeled as the 113, 114, and 115, and the three samples from the Cd-treated group were respectively labeled as the 116, 117, and 118. The aliquots in equal quantity from 6 samples were mixed and labeled as 119, and served as an intermediate to interact the samples.

Fig. S2 Related metabolomics pathways of differentially expressed proteins after Cd-exposure by KEGG classification. Each digital on the right of each bar indicates the number of proteins in each category. The letters A, B, E and F represent the 4 branches in KEGG pathways including Metabolism (A), Genetic Information Processing (B), Organismal Systems (E) and Human Disease (F).

Fig. S3 Effects of *L. plantarum* CCFM8610 on Cd-induced alterations in the levels of AchE (A), VIP (B), 5-HT (C), CGRP (D), and NO (E) in the jejunum of mice. The letters a and b mean the data of groups with different letters differ significantly (*P* < 0.05). The abbreviations of AchE, vip, 5-HT, CGRP and NO are defined in the legend of Figure 5.

Fig. S4 Effects of *L. plantarum* CCFM8610 on Cd-induced alterations in the expression of ERK (A), JNK (B) and bcl-2 (C) in the jejunum of mice. The letters a and b mean the data of groups with different letters differ significantly (*P* < 0.05). The abbreviations of ERK, JNK and bcl-2 are defined in the legend of Figure 6.
